# Effect of glazing and polishing on opalescence and fluorescence of dental ceramics

**DOI:** 10.1002/cre2.669

**Published:** 2022-10-17

**Authors:** Sedighe Sadat Hashemikamangar, Alireza Mahmoudi Nahavandi, Marzieh Daryadar, Sara Valizadeh, Mutlu Özcan

**Affiliations:** ^1^ Department of Operative Dentistry, Dental School Tehran University of Medical Sciences Tehran Iran; ^2^ Department of Color Imaging and Color Image Processing Institute for Color Science and Technology (ICST) Tehran Iran; ^3^ Department of Restorative Dentustry Tehran University of Medical Sciences Tehran Iran; ^4^ Department of Restorative School of Dentistry, Dental Research Center, Dentistry Research Institute, Tehran University of Medical Sciences Tehran Iran; ^5^ Division of Dental Biomaterials Center for Dental and Oral Medicine, Clinic for Reconstructive Dentistry, University of Zürich Zurich Switzerland

**Keywords:** dental ceramics, fluorescence, glazing, opalescence

## Abstract

**Objective:**

Tooth enamel has opalescence and fluorescence, which should be mimicked by esthetic dental restorations. The purpose of this study was to compare the effects of glazing and polishing on the opalescence and fluorescence of dental ceramics.

**Materials and Methods:**

Twenty‐four discs were fabricated of feldspathic, IPS e.max, zirconia, and Enamic ceramics with 10 mm diameter and 0.5 and 1 mm thicknesses (*n* = 12). Of the discs fabricated with 0.5 and 1 mm thicknesses, half of them were glazed and the remaining half were polished (*n* = 6). Opalescence was calculated as the difference in yellow‐blue (CIE ∆b*) and red‐green (CIE ∆a*) color axes between the transmitted and reflected colors. The fluorescence of specimens was measured by a novel technique. Data were analyzed using two‐way analysis of variance at a 0.05 level of significance.

**Results:**

In all groups (except for the Enamic ceramic), the mean opalescence of polished specimens (e.max = 2.704, feldspathic = 1.67, zirconia = 3.143) was higher than that of glazed specimens (e.max = 2.163, feldspathic = 1.016, zirconia = 2.690). The mean opalescence of glazed Enamic specimens (2.140) was higher than that of polished specimens (1.308). The fluorescence of glazed and polished specimens was not significantly different.

**Conclusion:**

Surface treatment (glazing/polishing) affects the opalescence, but not the fluorescence of dental ceramics evaluated in this study.

## 1 INTRODUCTION

It is imperative to achieve an excellent color match between the restoration and the adjacent natural teeth to meet patient expectations in terms of aesthetics. To achieve this goal, it is important to have comprehensive knowledge about tooth color as well as the color parameters of dental materials (Kim et al., 2016a, 2016b). Mimicking the natural tooth color is a major challenge for dentists and can only be achieved by excellent clinical and laboratory performance (Ishikawa‐Nagai et al., 2010). Esthetic dental restorations should have optical properties similar to those of natural teeth in terms of translucency, opacity, fluorescence, and opalescence (Monteiro et al., 2012; Vanini, 1996). The significance of opalescence and fluorescence of restorative materials, in addition to the conventional color parameters of value, hue, and chroma, has been previously emphasized for achieving ideal dental restorations (Tabatabaei et al., 2019). The opalescent stones, the sky, and the tooth enamel have the same color when reflecting light while they have different colors when refracting light; this phenomenon is referred to as opalescence. Dental materials possessing opalescence should be selected for the fabrication of esthetic dental restorations to be able to mimic the natural tooth appearance (Cho et al., 2009). The human enamel is opalescent, and light emission in lower wavelengths in the visible spectrum results in a bluish tint in the reflected color and an orange/brownish tint in the transmitted color (Nohl et al., 2002). Light emission occurs due to small particles in the visible light spectrum. The opalescence of dental materials is determined by the opalescence parameter, which is the difference in transmitted and reflected colors in yellow‐blue (CIE ∆b*) and red‐green (CIE ∆a*) color axes (Kobashigawa & Angeletakis, 2001; Lee et al., 2005a, 2005b; Lee & Yu, 2007; Lee, 2016).

Natural teeth have a bluish fluorescence under ultraviolet (UV) light, which is responsible for the whiter and lighter appearance of the teeth under daylight (Björkman et al., 1991; Spitzer & Bosch Ten, 1976). The fluorescence of natural teeth results in distribution of absorbed energy in higher wavelengths such that the teeth are converted to a light source. Dentin irradiated with monochromic light at 365 nm wavelength creates fluorescence with a peak at 440 ± 10 nm (Meller & Klein, 2012). The fluorescence of dental materials is determined based on their color difference in the presence and absence of UV light in a spectrophotometer (Lee et al., 2005a; Lee et al., 2006).

The use of indirect esthetic dental restorations, such as porcelain restorations, has greatly increased in recent years. The reasons for this increase in demand may be the relatively high failure rate of direct composite restorations as well as their marginal discoloration, marginal chipping, and wear (El Zohairy et al., 2003).

Ceramic restorations can favorably mimic the tooth properties, and therefore, bring about favorable esthetic results. Moreover, they have excellent optical properties, allowing the transmittance and reflectance of light. Thus, ceramics can provide a color match with optical properties close to those of natural teeth (Wildgoose et al., 2004). At present, dental porcelain has several applications as a restorative material in all‐ceramic restorations, metal‐ceramic crowns, and fixed partial dentures due to its favorable esthetic properties, optimal durability and biocompatibility, and satisfactory mechanical properties (El Zohairy et al., 2003).

However, some surface modifications are often required for occlusal adjustment, correction of the contour and finish line of ceramic restorations, enhancement of their esthetic appearance, and ultimate smoothness of porcelain restorations (Al‐Wahadni & Martin, 1998). The effects of several polishing techniques have been previously investigated for this purpose, and polishing has been recommended as an alternative to glazing for ceramic restorations (Goldstein, 1989; Haywood et al., 1988; Patterson et al., 1991; Scurria & Powers, 1994; Sethi et al., 2013). The surface quality, in terms of homogeneity and smoothness, is an important parameter affecting the color of restorations, because smoother surfaces can serve as a mirror and reflect a greater portion of light compared with rougher surfaces (Jung et al., 2004; Obregon et al., 1981). A search of the literature by the authors yielded one previous study on the effect of polishing on the opalescence of ceramics, reporting that glazing of monolithic zirconia porcelain significantly decreased its opalescence, compared with polishing (Kim et al., 2016a, 2016b). Another study evaluated the effect of different polishing systems on the color and surface texture of feldspathic porcelain. They conduced that different polishing techniques yielded a surface smoothness comparable to the glazing technique. Also, the color change (ΔE*) caused by all polishing techniques was within the clinically acceptable range (Sarac et al., 2006).

To the best of the authors' knowledge, no previous study has assessed the effect of polishing on the fluorescence of dental ceramics. Studies on the effects of polishing on the opalescence of dental ceramics are also limited, and further investigations are required on this topic.

Volpato et al. (2018) evaluated the fluorescence of natural teeth and restorative materials in a systematic review and highlighted the gap in information regarding the fluorescence of restorative materials, particularly dental ceramics. Thus, further studies are required on the opalescence and fluorescence of composite resins and ceramics, since the existing literature is not sufficient on this topic. Accordingly, this study aimed to assess the effects of glazing and manual polishing on opalescence and fluorescence of dental ceramic specimens fabricated with 0.5 and 1 mm thicknesses.

## 2 MATERIALS AND METHODS

This in vitro, experimental study evaluated four types of ceramics (due to their different composition) including VM9 feldspathic ceramic (A2 shade; Vita), IPS e.max ceramic (A2 shade, HT, Ivoclar), zirconia ceramic (A2 shade, Kerox Zircostar), and Enamic hybrid ceramic (A2 shade, Vita). Table 1 summarizes the characteristics of ceramics evaluated in this study.

**Table 1:** Characteristics of the ceramics evaluated in this study

Ceramic type	Composition
Feldspathic	15%–25% quartz leucite, pottasium feldspar, NAlSi_3_O_3_, KAlSi_3_O_3_, pigments, metal oxides
IPS e.max	SiO_2_, Li_2_O, K_2_O, P_2_O_5_, ZrO_2_, ZnO
Zirconia	ZrO_2_, Y_2_O_3_, Al_2_O_3_, SiO_2_, Fe_2_O_3_, Na_2_O
Enamic	SiO_2_, Al_2_O_3_, Na_2_O, K_2_O, B_2_O_3_, CaO, TiO_2_, PMMA

The minimum sample size was calculated to be 3 in each group according to a study by Della Bona et al. (2014) assuming *α* = .50, *p* = .2, mean standard deviation of 0.39, and the effect size of 1.04 using one‐way analysis of variance (ANOVA) power analysis feature of PASS 11 software.

Twenty‐four disc‐shaped specimens were fabricated from each ceramic type in two subgroups measuring 10 × 0.5 mm and 10 × 1 mm (*n* = 12). Each subgroup was then randomly divided into two smaller groups (*n* = 6) for glazing and polishing. The specimens then underwent baseline color assessment.

### 2.1 Preparation of specimens

#### 2.1.1 Feldspathic ceramic

Cylindrical silicon molds with 0.5 and 1 mm thicknesses and 10 mm diameter were used for the purpose of standardization. The porcelain powder was mixed with distilled water according to the manufacturer's instructions and applied to the mold. Excess water was removed by vibration. The porcelain was condensed in the mold, and after removal from the silicon mold, the ceramic was sintered according to the manufacturer's instructions as follows: 80 mbar vacuum was started at 450°C and ended at 919°C. The ceramic was heated and the temperature was increased at a rate of 55°C/min until reaching 920°C. The ceramic remained in this temperature for 90 s.

#### 2.1.2 IPS e.max

A wax pattern was designed with the desired dimensions using a computer‐aided design (CAD) machine. It was then transferred to a milling machine and the discs were milled with the desired dimensions. The wax patterns were then sprued and flasked using a 100‐g flask. The flask was heated to 700°C to evaporate and eliminate the wax pattern. Next, the ceramic ingot was added and sintered in a furnace. After heating to 910°C under vacuum, the plunger injected the ingot into the sprue until full. After compression and cooling, the gypsum particles were removed by sandblasting with 100 µ aluminum oxide particles at 2.5 bar pressure. The specimens were then immersed in Invex liquid and then in an ultrasonic bath for 4 min. They were then rinsed and dried, and impurities were removed by sandblasting with 100 µ aluminum oxide particles at 2.5 bar pressure. Next, the sprues were cut with a diamond disc under water coolant. Eventually, the specimens were sintered in a furnace under an 80 mbar vacuum starting at 410°C and ending at 725°C. The rate of temperature rise was 60°C/min until reaching 730°C. The ceramic remained at 730°C for 1 min.

#### 2.1.3 Zirconia

Ceramic specimens with the desired dimensions were designed by a CAD machine. The zirconia blank was placed in the milling machine and the discs were milled with the desired dimensions. After milling, the models were separated from the blank by a diamond bur. The dust was removed by air spray and they were placed on a plate for sintering in a furnace. They were then sintered at 1500°C for 120 min.

#### 2.1.4 Enamic

Ceramic specimens with the desired dimensions were designed in a CAD machine. The blocks were then placed in a milling machine and discs with the desired dimensions were milled. To ensure standardization of specimens in terms of thickness, the dimensions of all discs were measured by a caliper.

### 2.2 Polishing

The zirconia, IPS e.max, and feldspathic ceramic specimens were polished using a Panther polishing kit (Dental Direkt GmbH). The Enamic hybrid ceramic specimens were polished using Vita Enamic Polishing Set (Vita Zahnfabrik).

### 2.3 Glazing

The IPS e.max specimens were glazed using the powder and liquid provided by the manufacturer as follows: The powder and liquid were mixed and applied to the surface of the specimens. The specimens were then sintered in a furnace with a starting temperature of 450°C and a temperature rise of 60°C/min until reaching 725°C. The specimens were kept at this temperature for 1 min and were then allowed to cool down.

For the feldspathic and zirconia ceramic specimens, glazing was performed using the powder and liquid provided by the manufacturers as follows: The powder and liquid were mixed and applied to the surface of the specimens. The specimens were then sintered in a furnace with a starting temperature of 450°C and a temperature rise of 60°C/min until reaching 830°C. The specimens were kept at this temperature for 1 min and were then allowed to cool down.

For Enamic specimens, the special glaze liquid (VITA ENAMIC GLAZE) was applied and polymerized for 10 s.

### 2.4 Color assessment

The reflectance and transmittance spectra of specimens were measured by a spectrophotometer (CS2000; Konica Minolta). The color parameters of specimens in CIE L*a*b* color space were measured by CS10‐W spectrophotometer software under D65/2^0^ observation condition. For the calculation of opalescence, the specimens were fixed to a jig in the reflectance mode against a white tile. The device was calibrated with the white tile. Next, the reflectance spectrum of the specimen was measured. In the transmittance mode, the device was calibrated with a bulb light. The specimen was then placed against the bulb and the energy transmitted from the specimen to the device was measured. The ratio of the measured energies in calibration and from the specimen was calculated to measure the transmittance of the respective specimen.

For the calculation of fluorescence, the color of specimens was measured against a white background (a standard white reference) in the reflectance mode in the presence and absence of 100% UV light. For this purpose, first, the UV light source was turned off and the device was calibrated in presence of an incandescent light bulb and then the reflectance of the specimen was measured. Next, the UV light source and the incandescent light bulb were both turned on, and the device was calibrated with the white tile with both lights on. Then, the reflectance of the specimen was measured again. The measurements were repeated twice and the mean values were calculated and used for statistical analyses.

The fluorescence was calculated as the color difference (∆E*_ab_) in the presence and absence of UV light, using the formula below. In this formula, 0 and 100 values indicate the presence of 100% UV light and the absence of UV light in the standard CIE device, respectively (Lee, 2016).

$$FL+{\left[{\left(CIE\;L_{100}^{*}-CIE\;L_{0}^{*}\right)}^{2}+{\left(CIE\;a_{100}^{*}-CIE\;a_{0}^{*}\right)}^{2}+\left(CIE\;b_{0}^{*}\right)\right]}^{0.5}$$

The opalescence was calculated using the formula below where T and R indicate the transmittance and reflectance modes, respectively (Kobashigawa & Angeletakis, 2001; Lee et al., 2005a, 2005b; Lee & Yu, 2007):

$$OP={\left[{\left(CIE\;a_{T}^{*}-CIE\;a_{R}^{*}\right)}^{2}+{\left(CIE\;{b_{T}}^{*}-CIE\;{b_{R}}^{*}\right)}^{2}\right]}^{1/2}$$

### 2.5 Assessment of opalescence and fluorescence of different ceramics

**Figure 1:**
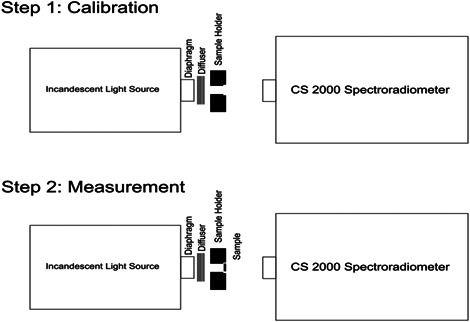
Measuring the transmittance

A spectroradiometer (CS‐2000; Konica Minolta) was used for measurement of the reflectance and transmittance of the specimens. For measurement of transmittance, first, an incandescent light source was used powered by a constant power supply. In front of the power supply, a paper was folded to achieve the ideal diffusion of light. Next, a black holder, made of plexiglass by a laser cutting machine, was used to hold the specimens. Figure 1 shows the measurement of transmittance by a spectroradiometer.

**Figure 2:**
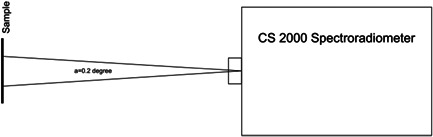
Measurement of color parameters at the center of specimen using a spectroradiometer

The transmittance of the specimens was read with the device angle adjusted at 0.2°. Considering the 80 cm distance between the specimen and the spectroradiometer, a circle with a 2.8 mm diameter at the center of the specimen was measured as shown in Figure 2.

**Figure 3:**
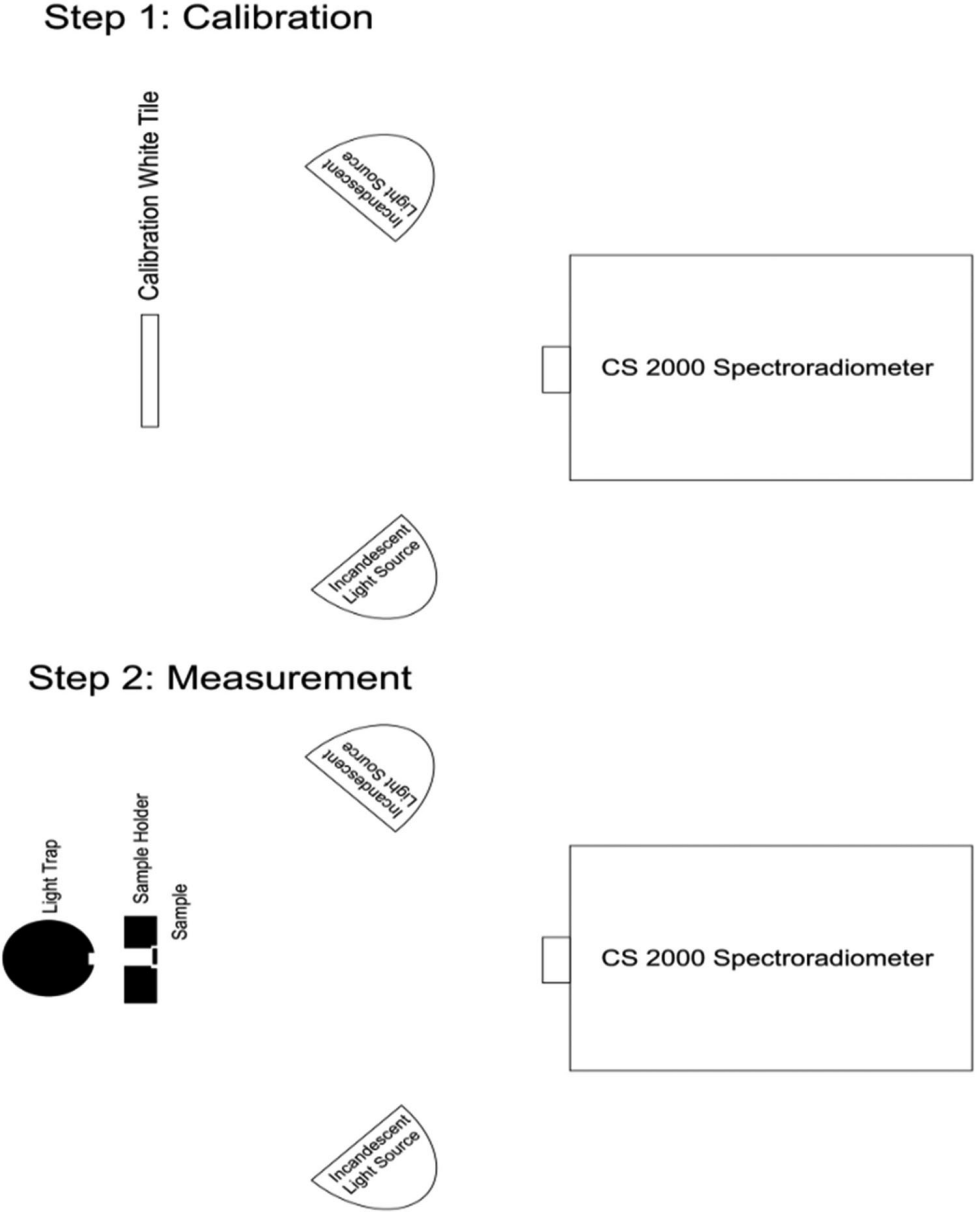
Measurement of reflectance by a spectroradiometer

For measurement of reflectance, two incandescent light sources were used to illuminate the specimens at a 45° angle. The lamps were lit using a power source. The device was then calibrated with a white tile. Next, the calibration white tile was removed and the specimens fixed to the holder were placed at the location of the tile. Also, since the specimens were semi‐transparent, an optical trap was placed behind them to prevent the return of light reflection after passing through the specimen. The reflectance of the specimens was then read. Figure 3 shows the measurement of reflectance by a spectroradiometer.

The opalescence of specimens was calculated using the difference in their chromaticity in transmittance and reflectance modes using the formula below:

$$op={\left({\left(\Delta a^{*}\right)}^{2}+{\left(\Delta b^{*}\right)}^{2}\right)}^{0.5}$$

In contrast to reflectance and transmittance, fluorescence is a function of the intensity of radiation. Even in spectrophotometers, standard fluorescent tiles are used in the background for the measurement of the reflectance of fluorescent specimens. Aside from the intensity of radiation, the excitation wavelength also affects the fluorescence. Generally, the efficiency of excitement varies depending on the wavelength. For this purpose, the fluorescence of specimens should be measured under standardized conditions. One important parameter in this respect is the distribution of UV spectrum of daylight, under which, the restored tooth is often viewed. Although logical, it is almost impossible to perform. Another solution is to use excitation at the maximum wavelength in equal intensity. For this purpose, the efficiency of emission of different wavelengths should be measured before excitation. Since the fluorescent reflectance should be compared with that of the specimen without UV excitation, the reflectance of the specimen in presence of UV excitation at the maximum wavelength was subtracted from that of the specimen in absence of UV excitation and was used for statistical analysis. A photoluminescence device (LS55, Perkinelmer) was used to find the maximum excitation wavelength.

Primary assessments have shown that the maximum emission wavelength of fluorescent specimens in presence of UV excitation occurs at 430–440 nm wavelengths. Due to the proximity of these wavelengths, the perceived color change by the human eye is almost the same, and thus, the difference between the maximum emission in the presence and absence of UV excitation was calculated as the fluorescence parameter.

**Figure 4:**
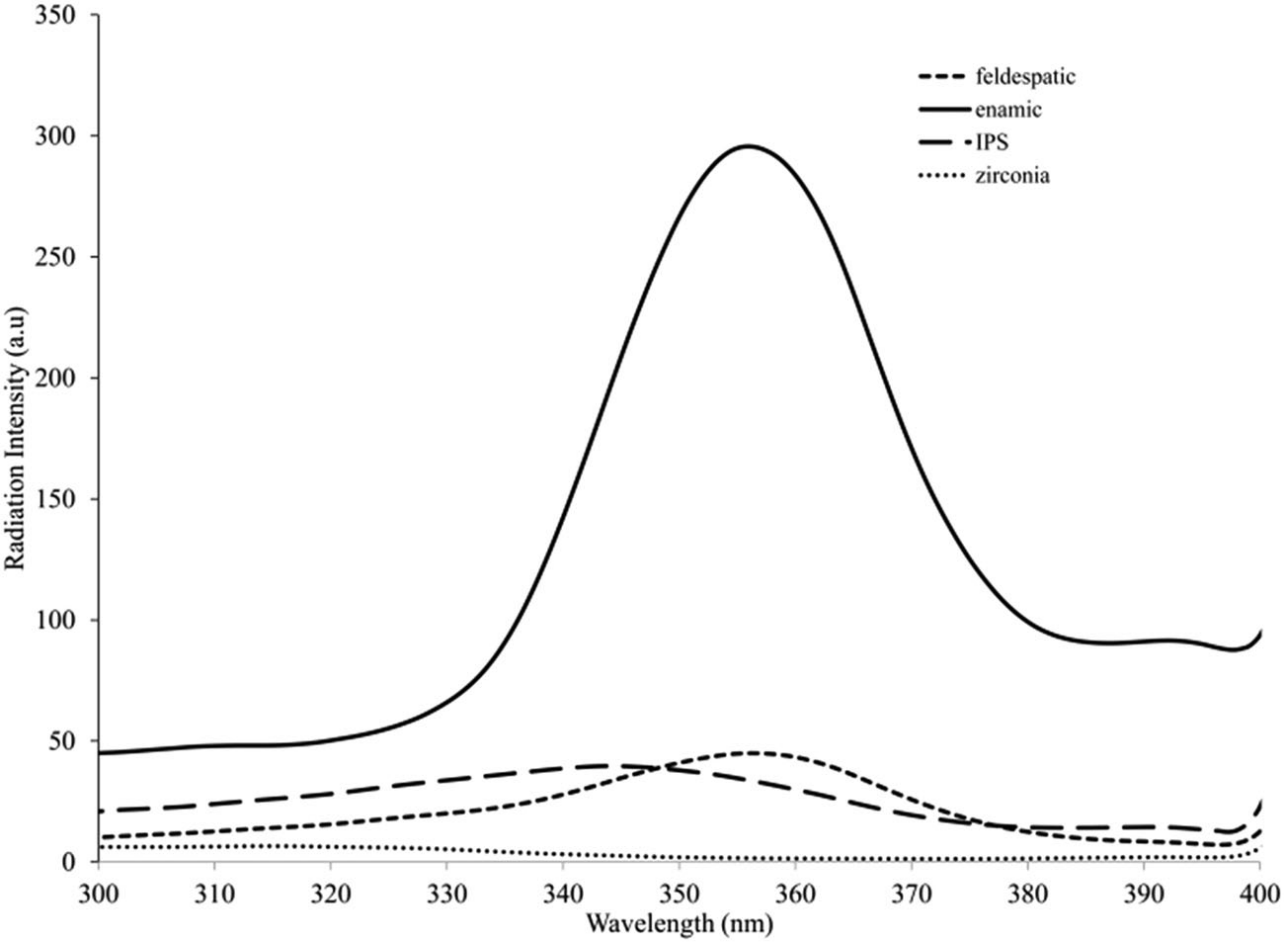
Emission efficiency of IPS e.max, feldspathic porcelain, Enamic, and zirconia ceramics

As shown in Figure 4, the zirconia ceramic was excluded from the fluorescence measurement due to the absence of emission efficiency.

Considering the equal maximum wavelengths of feldspathic, IPS e.max, and Enamic ceramics, light‐emitting diodes (LEDs) with 360 nm wavelength were used. Seven 1‐W LEDs were mounted on a board in series and lit by a 7‐W LED driver. Using a potentiometer, the intensity of LEDs was adjusted such that the maximum reflection of specimens did not exceed 300%, which was the maximum power of the measuring device.

**Figure 5:**
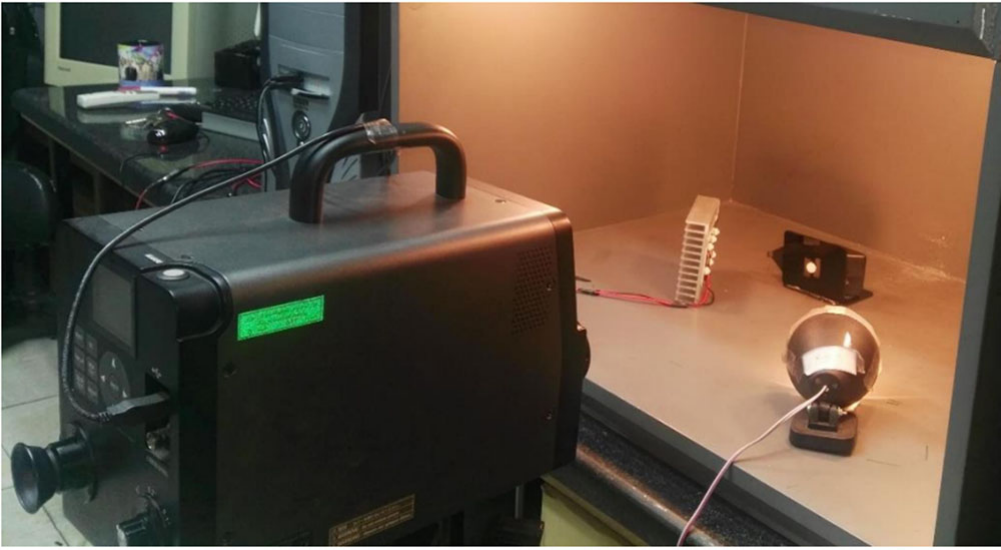
Florescent measurement by use of an incandescent light bulb with zero UV light. UV, ultraviolet

**Figure 6:**
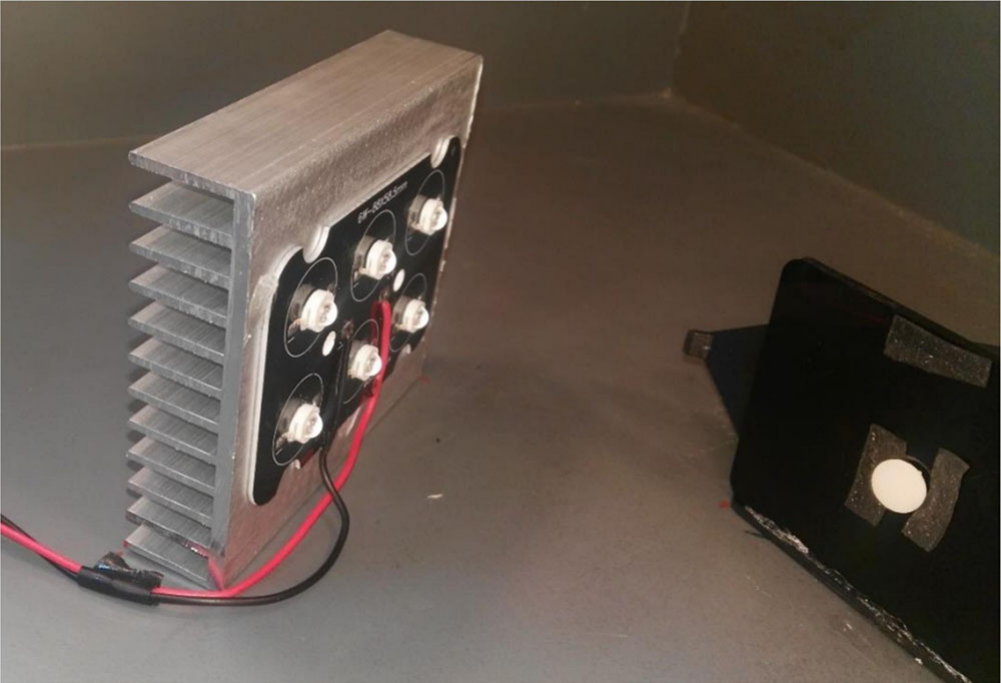
LEDs were added to the incandescent light bulb. LED, light‐emitting diode

Fluorescence was measured in two modes. Following the use of an incandescent light bulb with zero UV light and measurement of reflectance under this light as shown in Figure 5, the LEDs were added to the incandescent light bulb, the device was recalibrated with a white tile, and the reflectance was measured again as shown in Figure 6.

## 3 RESULTS

### 3.1 Assessment of fluorescence

Three‐way ANOVA was applied to assess the effects of type of ceramic, type of surface treatment, and thickness of specimens on fluorescence. Type of ceramic (*p* = .000) and thickness of specimens (*p* = .007) significantly affected the fluorescence; while, the effect of type of surface treatment on fluorescence was not significant (*p* = .384). Assessment of the interaction effects at the assessed level of confidence interval revealed that the interaction effects of the aforementioned three parameters were not significant on fluorescence (*p* > .05).

### 3.2 Assessment of opalescence

Three‐way ANOVA was applied to assess the effects of type of ceramic, type of surface treatment, and thickness of specimens on opalescence. Type of ceramic had a significant effect on opalescence (*p* = .000); while, type of surface treatment (*p* = .486) and thickness (*p* = .211) had no significant effect on opalescence. Assessment of the interaction effects at the assessed level of confidence interval revealed that the interaction effects of type of ceramic and thickness (*p* = .000), and type of ceramic and type of surface treatment (*p* = .000) were significant on opalescence. Thus, a univariate analysis was carried out.

### 3.3 Comparison of opalescence of different ceramic types (univariate analysis and pairwise comparisons for the interaction effect of type of ceramic and type of surface treatment)

Table 2 shows the mean opalescence of ceramics after the two surface treatments (polishing and glazing). As shown, in all groups (except for the Enamic ceramic), the mean opalescence of polished specimens (e.max = 2.704, feldspathic = 1.167, zirconia = 3.143) was higher than that of glazed specimens (e.max = 2.163, feldspathic = 1.016, zirconia = 2.690). In Enamic specimens, the mean opalescence of glazed specimens (2.140) was higher than that of polished specimens (1.308).

**Table 2:** Mean opalescence of ceramics after the two surface treatments (polishing and glazing)

Dependent variable: Opalescence					
Ceramic type	Treatment	Mean	Standard error	95% Confidence interval	
				Lower bound	Upper bound
e. max	Glazing	2.163	0.158	1.848	2.478
	Polishing	2.704	0.158	2.389	3.020
Enamic	Glazing	2.140	0.158	1.824	2.455
	Polishing	1.308	0.158	0.992	1.623
Feldspathic	Glazing	1.016	0.158	0.700	1.331
	Polishing	1.167	0.158	0.852	1.482
Zirconia	Glazing	2.690	0.158	2.374	3.005
	Polishing	3.143	0.158	2.827	3.458

**Diagram 1:**
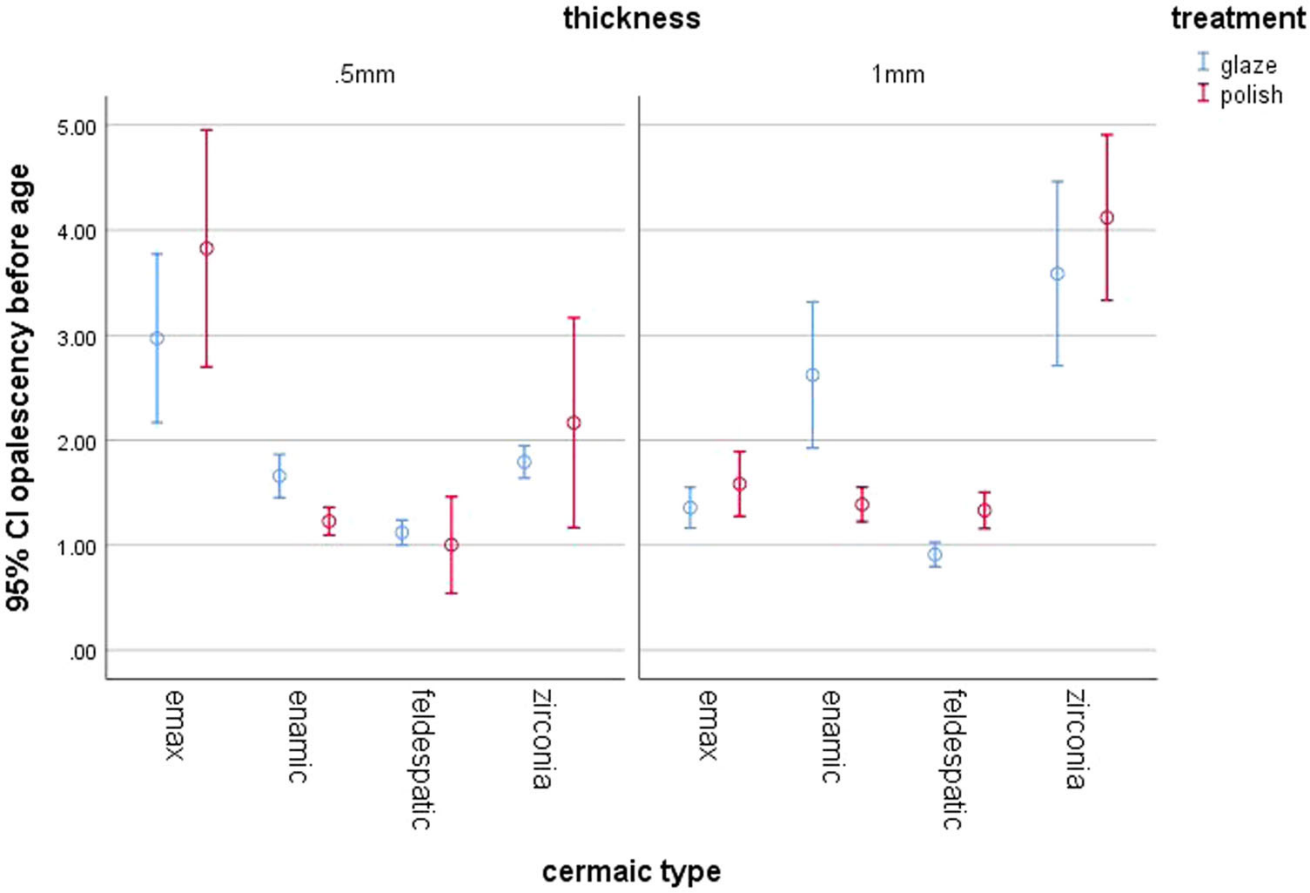
Opalescence of different ceramic types in glazed and polished subgroups

Table 3 shows pairwise comparisons of opalescence of ceramics in different surface treatment subgroups. As shown, among the glazed specimens, e.max showed significantly higher opalescence than feldspathic specimens (*p* = .000). The opalescence of Enamic specimens was significantly higher than that of feldspathic specimens (*p* = .000). Also, the opalescence of zirconia specimens was significantly higher than that of feldspathic specimens (*p* = .000). Among the polished specimens, e.max showed significantly higher opalescence than Enamic specimens (*p* = .00). The opalescence of e.max was significantly higher than that of feldspathic specimens (*p* = .000). The opalescence of Enamic specimens was significantly lower than that of zirconia specimens (*p* = .000). The opalescence of feldspathic specimens was significantly lower than that of zirconia specimens (*p* = .000; Diagram 1).

**Table 3:** Pairwise comparisons of opalescence of ceramics in different surface treatment subgroups

Pairwise comparisons							
Dependent variable: Opalescence							
Treatment	(I) Ceramic type	(J) Ceramic type	Mean difference (I–J)	Standard error	Sig.^b^	95% Confidence interval for difference^b^	
						Lower bound	Upper bound
Glazing	e.max	Enamic	0.024	0.224	1.000	−0.582	0.630
		Feldspathic	1.148*	0.224	0.000	0.542	1.754
		Zr	−0.526	0.224	0.128	−1.132	0.080
	Enamic	F	1.124*	0.224	0.000	0.518	1.730
		Zr	−0.550	0.224	0.098	−1.156	0.056
	F	Zr	−1.674*	0.224	0.000	−2.280	−1.068
Polishing	e.max	Enamic	1.397*	0.224	0.000	0.791	2.003
		F	1.537*	0.224	0.000	0.931	2.143
		Zr	−0.438	0.224	0.323	−1.044	0.168
	Enamic	F	0.141	0.224	1.000	−0.465	0.747
		Zr	−1.835*	0.224	0.000	−2.441	−1.229
	F	Zr	−1.976*	0.224	0.000	−2.582	−1.370

*Note*: Based on estimated marginal means.

Abbreviations: F, Feldspathic; Sig, Significance; Zr, Zirconia.

*Represent the significant difference (p Value = .05) between ceramics' opalascence.

b Significance is equal to .05.

## 4 DISCUSSION

### 4.1 Fluorescence

This study revealed a significantly descending order of fluorescence in Enamic, feldspathic, and e.max ceramics. To date, limited studies have assessed and compared the fluorescence of dental ceramics. Uranium was used as a luminophore in the first fluorescent ceramic. In combination with cerium oxide in porcelain, it resulted in generation of blue‐white fluorescence, which was highly similar to the fluorescence of natural teeth. Nonetheless, since uranium salt and its isotopes are highly radioactive, they are banned for use in dentistry and have been replaced with other luminescents such as ytterbium, cerium, terbium, and europium, which are rare earth oxides with strong fluorescence, resembling that of natural teeth, when exposed to UV light (Volpato et al., 2018).

The intensity of fluorescence emission is strongly correlated with the type and composition of ceramics. The emission and excitation spectra also depend on factors such as the ceramic composition, the crystalline matrix, and the sintering protocol (Volpato et al., 2018). Ecker et al. (1985) showed that repetition of the sintering cycle, similar to increasing the sintering temperature, decreased the intensity of fluorescence emission. This can explain the low fluorescence of e.max and feldspathic ceramics, compared with Enamic ceramic.

It should be noted that the high fluorescence of a dental material does not necessarily indicate its superiority because a ceramic may have high fluorescence but shift the restoration color to blue, which would create an unesthetic appearance. Dental ceramics should have a fluorescence similar to that of natural teeth.

According to Kin et al, (Kim et al., 2009) some zirconia ceramics are nonfluorescent. This statement was in agreement with our results since zirconia ceramic did not have fluorescence in our study. Several materials were introduced to the dental market to improve the optical behavior of this ceramic such as zirconia liner (for application on the surface) and fluorescent liquid (in which, the zirconia is immersed before sintering) (Kim et al., 2009).

No significant difference was noted in our study groups regarding the effect of different surface treatments (glazing and polishing) on fluorescence. Since the surfaces were only smoothened by polishing, no change in fluorescence seems logical. In glazing, however, a transparent film is applied on the surface. If the glazing material interacts with the surface and absorb UV light, it would prevent the UV from entering the system. Thus, the excitation and subsequently the fluorescence would decrease. If the film is UV‐absorbent, a yellow tint is observed on the glazed surface. However, if the interaction between the radiated UV light and the absorbed blue light is insignificant, it would have no significant effect on fluorescence. In this study, the glaze material was transparent. Thus, the absorption of blue light was insignificant and therefore, glazing had no significant effect on fluorescence. The effect of different surface treatments on fluorescence has not been investigated before to compare our results.

### 4.2 Opalescence

The mean opalescence had a decreasing order in zirconia, e.max, Enamic, and feldspathic ceramics. Regarding the effect of different surface treatments on opalescence, the glazed zirconia, e.max and feldspathic ceramic specimens showed lower opalescence than polished specimens; this difference was significant for e.max and zirconia ceramics (except for feldspathic ceramic). Our results were in agreement with those of Kim et al. (2016b) According to their study, glazing decreased the opalescence of monolithic zirconia, compared with polishing.

Here a question is raised why glazing of e.max, zirconia, and feldspathic ceramics would decrease their opalescence compared with polishing, while this trend was different for Enamic specimens. If the glaze applied on the surface has a yellow tint, it would absorb the blue light and prevent it from entering the opalescent medium until scattering, and thus, the opalescence behavior would decrease. Therefore, it may be concluded that the glaze film applied on the surface has a selective behavior in absorption, which interferes with the opalescence of the ceramic and decreases the difference between the reflectance and transmittance behaviors of the ceramic. Regarding Enamic ceramic, the interaction of the glaze material of Enamic is different from that of other ceramics, and therefore, it shows different behavior, which can be attributed to the different composition of the glaze material of Enamic ceramic. In fact, the glaze material of Enamic ceramic has an opposite function and intensifies the blue light.

## 5 CONCLUSION

Under the limitation of this study, Surface treatment (glazing/polishing) affects the opalescence, but not the fluorescence of dental ceramics evaluated in this study.

## AUTHOR CONTRIBUTIONS

**Sedighe Sadat Hashemikamangar**: contribution in study idea, study design, data collection, preparing article, revising article. **Alireza Mahmoudi Nahavandi**: contribution in study design, data collection, preparing article, revising article. **Marzieh Daryadar**: contribution in study design, data collection, preparing article. **Sara Valizadeh**: contribution in study idea, study design, data collection, preparing the article, revising article. **Mutlu Özcan**: contribution in study idea, study design, revising article.

## CONFLICT OF INTEREST

The author declares no conflict of interest.

## Data Availability

The data are available for any further consideration.
